# Robotic Revolution in Surgery: Diverse Applications Across Specialties and Future Prospects Review Article

**DOI:** 10.7759/cureus.52148

**Published:** 2024-01-12

**Authors:** Maryam Fairag, Rahf H Almahdi, Abeer A Siddiqi, Fares K Alharthi, Badran S Alqurashi, Naif G Alzahrani, Ahmed Alsulami, Rayan Alshehri

**Affiliations:** 1 Family Medicine, Makkah Healthcare Cluster, Makkah, SAU; 2 General Surgery, Jazan University, Jazan, SAU; 3 General Physician, Ministry of Health, Jeddah, SAU; 4 Cardiac Surgery, King Abdulaziz Specialist Hospital, Taif, SAU; 5 Faculty of Medicine, King Abdulaziz University Hospital, Jeddah, SAU; 6 Family Medicine, King Fahad Armed Forces Hospital, Jeddah, SAU; 7 Medicine, King Abdulaziz University Hospital, Jeddah, SAU; 8 Emergency Medicine, King Abdulaziz University Hospital, Jeddah, SAU

**Keywords:** cardiothoracic and vascular surgery, robotic assisted, urology, da vinci surgical system, robotic surgery

## Abstract

Robotic technology has transformed the field of surgery significantly. Since its inception in the 1970s, robotic surgery has advanced tremendously. The utilization of robotic systems, such as the da Vinci Surgical System, has become increasingly prevalent in minimally invasive procedures. These interventions offer enhanced precision, dexterity, and visualization. In general surgery, robotics has facilitated complex procedures, leading to reduced morbidity and shorter hospital stays. In urology, the robotic platform has revolutionized prostatectomies and other intricate interventions, demonstrating superior outcomes compared to traditional approaches. Orthopedic surgery has embraced robotics for precise joint replacements and spinal procedures. In pediatric surgery, the application of robotics has enabled intricate surgeries with reduced invasiveness and faster recovery times. Furthermore, the integration of artificial intelligence with robotic systems has paved the way for personalized treatment plans and data-driven decision-making. Despite these advancements, challenges such as cost and training persist. As robotic technology continues to evolve, its potential applications extend beyond current boundaries. This review aims to provide insights into the multifaceted impact of the robotic revolution in surgery and the exciting possibilities that lie ahead.

## Introduction and background

The healthcare landscape of the future is expected to be highly complex, as there is a rapidly increasing aging population that suffers from multiple chronic diseases [1]. In such a scenario, healthcare systems need to manage the long-term complications of aging patients. Similarly, the demand for surgical care will also continue to grow. In recent years, a significant advancement has been seen in the surgical arena with the integration of robotics into surgical practice [2]. The marriage of cutting-edge technology and the delicate art of surgery has had a profound impact on patient outcomes. The initial concept of robotic surgery originated in the 1970s, when the United States Defense Research and Advanced Projects Agency and the National Aeronautics and Space Administration (NASA) tried to create a system that aimed to enable remotely controlled surgery [3]. In 1985, the Programmable Universal Machine for Assembly 560 (PUMA 560) was the first program that was used in neurosurgical biopsy [4].

One of the hallmarks of robotic surgery is the da Vinci Surgical System, a pioneering platform that has become synonymous with robotic-assisted procedures. In 1999, the da Vinci surgical system was introduced by Intuitive Surgical, which was later approved by the United States Food and Drug Administration (FDA) in 2000 [5]. After its approval, it became a commonly employed system in robotic-assisted laparoscopic abdominal procedures. Since then, various newer generations of da Vinci systems have been developed [6]. The first da Vinci robotic system had three arms with an endoscope, which was upgraded to four arms. The latest da Vinci Xi platform was released in 2014 [7]. The most common parts of any robotic system are the arm and console. One arm is equipped with a camera, while the other arm carries the surgical instruments. The console provides the operator with a precise, magnified, and high-quality view of the surgical site. The surgeon takes the lead, directing other team members to assist during the operation [8]. This level of precision has redefined the boundaries of what is achievable in surgery, particularly in complex and minimally invasive procedures.

The impact of robotic surgery is not confined to a specific medical discipline but extends across a spectrum of specialties. From general surgery to urology, gynecology, and even cardiac surgery, the versatility of robotic systems has transcended the confines of traditional surgical approaches. Surgeons now wield the ability to navigate intricate anatomical structures with enhanced vision and unparalleled precision [9]. As a result, patients often experience less pain, fewer complications, and faster returns to their daily lives. While the benefits of robotic surgery are undeniable, the integration of this technology has not been without its challenges. The initial costs associated with acquiring and maintaining robotic systems have been a deterrent for some healthcare institutions. An investigation by Tedesco et al. revealed that at least 349 annual procedures are required to reach the break-even point for robotic surgery [10]. As the robotic revolution in surgery continues to unfold, addressing these challenges will be paramount to ensuring equitable access to this transformative technology.

Robotic-assisted surgery has transformed surgical practices around the world, going beyond traditional limits. Surgeons from different countries have adopted this technology due to its accuracy and improved visualization. It is applicable to a wide range of medical specialties, providing patients with reduced pain, fewer complications, and faster recovery times [3,4]. Despite the initial costs, healthcare institutions are striving to ensure equal access to this transformative technology. Ongoing research and development are pushing the boundaries, with the potential for further integration of artificial intelligence and autonomous surgical systems. Robotic-assisted surgery is shaping the future of surgical practice and enhancing patient outcomes on a global scale [7]. The advent of robotic-assisted surgery represents a paradigm shift that transcends traditional boundaries. This review article uncovers the profound impact of the robotic revolution in surgery, exploring the evolution, current state, and future prospects of this transformative field.

## Review

Application in general surgery

The field of general surgery and its sub-specialties have been significantly transformed by the introduction of robotic technology, allowing for minimally invasive procedures. Sub-specialties such as colorectal, hepatobiliary, pancreatic, gastric oncologic, bariatric, foregut, pediatric, endocrine, and hernia surgery have adopted robotic surgery and conducted thorough research to assess its impact on patient outcomes [11]. Robotic systems have proven highly effective in colorectal cancer surgery, particularly in challenging procedures like the total excision of the mesorectum and complete mesocolon excision. In such surgeries, the robot aids in tasks such as vascular dissection, intracorporeal anastomoses, and lymphadenectomy, especially in complex anatomical spaces like areas near vital vascular structures or the lateral side walls of the pelvis. In many medical centers, the use of robotic assistance has now become the standard approach for rectal resections among colorectal surgeons. This adoption reflects the increased success and benefits offered by robotic surgery in these technically demanding colorectal procedures [12].

A major concern about the widespread applicability of robotic assistance in colorectal surgery has been the high cost of the procedures. However, a significant amount of evidence has consistently highlighted the undeniable benefits of robotic surgery, particularly in left colectomies and various rectal procedures, surpassing even the capabilities of advanced 3-D laparoscopic systems [13]. Furthermore, robotic-assisted surgery has the ability to overcome inherent weaknesses and achieve outcomes on par with traditional laparoscopy [14]. The advantages of robotic surgery in colorectal resections include reduced blood loss, shorter hospital stays, quicker restoration of bowel function, favorable oncological outcomes, and a diminished rate of conversion to open surgery [15]. Similar findings have been shared in a meta-analysis by Trastulli et al. that compared robotic and laparoscopic colorectal resections. They revealed that robotic surgical procedures had a lower occurrence of perioperative complications and surgical site infections [16].

In another study, Liao et al. highlighted the effectiveness of robotic surgery in rectal cancer [17]. The findings indicated that robotic surgery demonstrated high efficacy and comparability with open surgery. Similar outcomes were observed in terms of oncologic results, lymph node yields, free margins, disease-free survival, and complication rates. The study found that there were no significant differences in surgery-related complications, oncologic clearance, disease-free survival, or overall survival between the two groups [17]. Although the operation duration was longer, the study suggests that increased surgical volumes could lead to improvements in this aspect. Another promising aspect of robotic rectal resections is the integration of Firefly™ technology. Firefly™ technology is particularly useful during the low ligation of the inferior mesenteric artery (IMA) pedicle. The advantages of robots in retroperitoneal and pelvic dissection play a crucial role in enabling precise lymphadenectomy around the IMA [18].

The use of robotics in bariatric surgery has been developing since Cadiere et al. first reported a case in 1999 [19]. Many specialists consider Roux-en-Y gastric bypass to be the most effective surgical procedure for severe obesity. Robotic surgery is now seen as an appealing technology that could enhance the performance of Roux-en-Y gastric bypass due to its well-documented advantages. In fact, it is the most extensively studied robotic bariatric procedure [20]. Sleeve gastrectomy is gaining popularity due to its low risk of complications, excellent outcomes, and perceived technical simplicity. However, there are certain unique aspects of sleeve gastrectomy that need to be considered, such as the potential for leakage along the long staple line and the need for precise and safe dissection in the left crus and hiatus area to mobilize the fundus. Robotic surgery, in comparison to laparoscopic surgery, offers the advantage of endo-wrist capability, which facilitates the dissection of the hiatus and allows for precise suturing of the staple line [21]. A systematic review by Cirocchi et al. revealed that robotic bariatric surgery is not limited to redo cases but is increasingly employed in primary procedures. This includes situations where surgeons utilize robotic assistance to create intracorporeal gastrojejunostomy or jejunojejunostomy anastomosis during Roux-en-Y gastric bypass or to address challenging gastric resections in sleeve gastrectomy. Furthermore, even in cases where stapling is chosen for anastomoses during Roux-en-Y gastric bypass, robotic technology facilitates the more efficient closure of enterotomies or gastrotomies [22]. 

Robotic surgery is widely applicable in the field of urology, with implications for procedures like prostatectomy, which is commonly performed to treat prostate cancer. The introduction of robotic systems has revolutionized prostatectomy by providing enhanced precision and three-dimensional vision. Extensive research has indicated that robotic-assisted prostatectomy offers several advantages over traditional open surgery, including reduced blood loss, shorter recovery periods, and improved functional outcomes [23]. Robotic surgery has emerged as a valuable alternative to traditional surgical approaches in various fields. Numerous studies have compared the outcomes of robotic surgery with those of traditional open surgery and laparoscopic surgery, revealing several advantages of the robotic approach. In terms of surgical precision and dexterity, robotic surgery offers superior capabilities due to its enhanced visualization, tremor filtration, and intuitive motion scaling [12]. 

Other fields of general surgery, such as hepatobiliary and pancreatic surgery, are also benefiting from robotic assistance. A study including 250 robotic pancreatic resections showed that robotic-assisted surgery was not only feasible for both oncologic and benign diseases but also had a low conversion rate [24]. However, it is important to keep in mind that robotic technology is merely a tool at the surgeon's disposal, with the ultimate responsibility lying in the hands of the surgeon [25]. The robotic platform provides surgeons with the ability to overcome various limitations encountered in laparoscopy, particularly when conducting a D2 lymphadenectomy [26]. The surgical robot has demonstrated its utility in tasks such as performing robotic-sewn anastomoses and navigating challenging dissections near the gastroesophageal junction and pyloric region. This assistance is particularly beneficial for procedures like total gastrectomies [27].

Applications in pediatric surgery

To this day, the standard procedure for the treatment of pediatric patients is minimally invasive surgery [28]. In comparison to adult robotic surgery, pediatric robotic surgery is swiftly gaining popularity in a wide spread of pediatric surgical subspecialties due to its unique challenges. The current applications of robotic surgery in pediatric patients include a variety of subspecialties, such as general and thoracic surgery, otolaryngology, urology, and surgical oncology. Robotic surgery is most commonly used in the urological specialty among the pediatric population [29]. Robotic surgery has demonstrated significant advantages, including enhanced capabilities, improved workplace efficiency, superior visualization, reduced surgeon fatigue, and a decrease in physiological tremors compared to laparoscopic and open surgery [30]. As the demand for precise surgical procedures and safety increased, surgeons increasingly embraced minimally invasive techniques, such as laparoscopy and thoracoscopy. These approaches offer various benefits, including reduced wound trauma, shorter hospital stays, improved visualization, decreased postoperative complications (such as wound infections or incisional hernias), minimized tissue damage, faster tissue healing, and quicker hospital discharge, resulting in enhanced cost-effectiveness [31]. While certain procedures have showcased the superiority of robotic surgery, overall, laparoscopic surgery has proven to be superior across a wider range of procedures. The significantly higher costs associated with robotic surgery have raised questions about its value [32]. With greater demand from both physicians and patients, robotic surgeries continue to grow, regardless of available outcomes and the costs of research.

Robotic urologic surgery in adults commenced with prostatectomy, followed by the first pediatric robot-assisted laparoscopic pyeloplasty (RALP) in 2002 [33]. By 2015, approximately 40% of pyeloplasty procedures on children in the United States were conducted using robotic assistance [34]. Numerous meta-analyses and systematic reviews on surgical outcomes following RALP have revealed shorter operative times (excluding docking time), reduced hospital stays, and comparable success rates when compared to open or laparoscopic procedures [35,36]. The application of robotic technology in general surgery for pediatric patients has garnered attention, although its prevalence is not as extensive as in pediatric urology. Meininger et al. documented their use of robotic-assisted laparoscopic techniques in performing Nissen fundoplication in 2001 [37]. Since then, fundoplication has emerged as one of the most frequently conducted robotic general surgeries in pediatric cases [38].

Applications in cardiothoracic surgery

Early in the new millennium, there was a growing interest in cardiac surgery for robotic and less invasive procedures. Because of their improved dexterity and three-dimensional (3D) endoscopic magnification, robotically assisted surgical systems have been used to boost the precision of minimally invasive and endoscopic surgery. Surgical telemanipulation has been expanded to additional cardiac procedures such as coronary revascularization, left ventricular lead implantation, congenital heart surgery, and aortic valve replacement due to the success of robotic mitral valve surgery [39]. Common robotic cardiothoracic procedures in pediatric patients include diaphragmatic hernia repair, lobectomy, excision of bronchogenic or mediastinal cysts, Heller's cardiomyotomy for achalasia, oesophagoplasty, and repair of oesophageal atresia [40].

Minimally invasive surgical techniques have seen major advancements with the introduction of robotic-assisted surgery. Over the past decade, robotic mitral valve surgery has emerged as the preferred procedure for mitral valve replacement and repair at specialist facilities worldwide due to its superior outcomes. Patients can benefit from shorter hospital stays and quicker recovery times, allowing them to return to normal activities sooner [41]. The primary objective of robotic mitral valve surgery is to emulate the high-quality outcomes of a traditional sternotomy-based mitral valve operation while employing a less invasive approach. Robotic procedures maintain the safety and effectiveness of the operation, offering clinical advantages such as reduced blood loss, a lower risk of incisional infection, and shorter hospital stays compared to sternotomy-based surgery [42,43]. A systematic review and meta-analysis, encompassing 14 studies and 6,341 patients (2,804 undergoing robotic mitral valve surgery and 3,537 undergoing sternotomy), compared early surgical outcomes [44]. The majority had degenerative mitral valve disease (94.6% in robotic surgery and 90.5% in sternotomy). The mitral valve repair rate was higher in the robotic group (93.8% vs. 71.0%). Although robotic surgery had longer aortic cross-clamp and cardiopulmonary bypass times, it was associated with a lower incidence of postoperative renal insufficiency [44]. While the safety and efficacy of standard or minimally invasive mitral valve surgery are commonly discussed, patient satisfaction is often influenced by factors such as postoperative pain, recovery, cosmetic results, and the time needed to return to normal activity [45]. Mitral valve surgery was compared in a head-to-head study between standard and robotic approaches. Both methods effectively improved the quality of life, with little difference at the two-year mark. However, robotic surgery demonstrated a faster recovery, enabling a quicker return to work and normal daily activities (33 days for robotic repair vs. 54 days for open repair). This early benefit is likely to significantly enhance patient satisfaction and alleviate the stress associated with the operation [46].

Standardized protocols for robotic single-vessel and double-vessel complete endoscopic coronary artery bypass grafting have also been developed for beating and non-beating hearts. While other cardiac procedures are at different stages of development, current results correspond with the main clinical trial outcomes for surgeries involving sternotomies [41]. Traditionally, coronary artery bypass grafting (CABG) was conducted through a conventional sternotomy with the assistance of a cardiopulmonary machine. The advent of the da Vinci system has introduced new robotic surgical approaches, including robot-assisted direct coronary artery bypass and totally endoscopic coronary artery bypass. In a comprehensive systematic review incorporating both comparative and noncomparative studies on robot-assisted direct coronary artery bypass and totally endoscopic coronary artery bypass the researchers identified acceptable and comparable perioperative mortality rates across all procedures. The authors conducted a thorough examination of intraoperative details and postoperative outcomes, recognizing the limitations of the existing clinical evidence. Despite these limitations, the authors concluded that the insights gleaned from their review should be considered a valuable benchmark for future studies in the field [47]. Hammal et al. conducted a meta-analysis comprising 13 studies, encompassing 11 primary investigations, comparing robotic coronary artery bypass (RCAB) with coronary artery bypass grafting (C-CABG) in seven studies, minimally invasive direct coronary artery bypass (MIDCAB) in three studies, and percutaneous coronary artery bypass (PA-CAB) in one study. Aggregate analyses indicated that RCAB exhibited lower rates of pneumonia or wound infection compared to C-CABG, along with a shorter length of stay in the intensive care unit compared to both C-CABG and MIDCAB [48].

Over the past two decades, robotic surgery has predominantly been employed for congenital issues, with limited literature available on the subject. Despite that, in the realm of adult congenital heart disease, there is a growing belief that robotic surgery presents a superior strategy compared to traditional minimally invasive cardiac surgery [48]. The robotic platform offers an exceptional perspective on the intricate anatomy often encountered in adult congenital cases. The capacity to maneuver the camera within the ventricles, coupled with superior image resolution, facilitates the repair of complex congenital tricuspid or mitral valves. It also allows for approaching a double-chambered right ventricle (DCRV) through the tricuspid orifice without necessitating ventricular incisions. Additionally, the robotic technique holds promise for addressing complex ventricular arrhythmias, commonly affecting adult patients with congenital heart defects [49]. Furthermore, even in cases of complex redo chest procedures frequently encountered in adult congenital cardiac patients, robotic cardiac surgery confers significant advantages in maintaining a genuinely minimally invasive technique. Notably, owing to the absence of thoracotomy incisions, robotic surgery minimizes postoperative pain, yields excellent cosmetic results, and enables these typically young patients to resume full activity more quickly [39].

Robotic surgery offers several advantages, including reduced invasiveness, immunity to surgeon hand tremors, improved cosmetic outcomes, and potential benefits such as decreased discomfort, fewer complications, reduced need for transfusions, and shorter hospital stays, although these latter advantages require further validation. On the flip side, drawbacks include extended operating times, heightened costs, uncertainty surrounding graft patency when performed by less experienced surgeons, hindered completion of revascularization, and, in certain studies, elevated mortality rates [50]. The safety and efficacy profiles of both surgical techniques for mitral valve surgery are satisfactory. There is currently little information on the relative performance of robotic mitral valve surgery RMVS and conventional sternotomy mitral valve surgery CSMVS, as there are only poor-quality studies with a moderate to severe risk of bias available. RMVS may, in certain patients, lead to a decrease in mortality as well as a shorter duration of stay in the ICU and hospital than CSMVS. Conversely, it is possible that CSMVS is linked to noticeably reduced cross-clamp and CPB times. To confirm these findings and evaluate the variations in mitral valve repair quality and postoperative quality of life between the two surgical techniques, high-quality studies utilizing randomized data are needed [44].

Table 1 lists the robotic surgeries performed in the literature.

**Table 1:** Descriptive characteristics of robotic-assisted surgeries: systematic review and meta-analysis of the last five years.

Author and Year	Robotic Surgery Name	Subspecialty	Average Surgery Duration	Average Post-Operative Hospital Stay
Guadagni et al. (2024) [51]	Robotic hepatectomy	Hepatobiliary and pancreatic surgery	500 min	12.2 days
Csirzó et al. (2023) [52]	Robotic-assisted laparoscopy	Gynecological robotic surgery	28.09 min	5.41 days
Cuk et al. (2022) [53]	Robot-assisted colon surgery	Colorectal robotic surgery	220 min	6.54 days
Coletta et al. (2021) [54]	Robotic hepatectomy	Hepatobiliary and pancreatic surgery	194–456 min	6.6 days
Safiejko et al. (2021) [55]	Robotic-assisted rectal cancer resection	Colorectal robotic surgery	N/A	8.0 ± 5.3 days
Kamarajah et al. (2021) [56]	Robotic hepatectomy	Hepatobiliary and pancreatic surgery	281 min	7 to 8 days
Marchand et al. (2021) [57]	Robotic-assisted gynecologic laparoscopy	Gynecological robotic surgery	N/A	1.25 days
Wang et al. (2021) [58]	Robotic hepatectomy	Hepatobiliary and pancreatic surgery	28.65 min	2.25 days
Mancino et al. (2020) [59]	Robotic total knee arthroplasty	Orthopedic surgery	88 min	N/A
Kamarajah et al. (2020) [60]	Robotic pancreatic duodenectomy	Hepatobiliary and pancreatic surgery	405 min	12 days
Wang et al. (2020) [61]	Robotic-assisted rectal surgery	Colorectal robotic surgery	265.1 min	7.83 days
Restaino et al. (2020) [62]	Robotic-assisted endometrial surgery	Gynecological robotic surgery	173 min	1.16 days
O’Sullivan et al. (2019) [63]	Robotic thymectomy	Robotic-assisted thoracic surgery	138.5 min	3.48 days
Ma et al. (2019) [64]	Robotic-assisted right colectomy	Robotic-assisted Colorectal surgery	200.61 ± 84.50 min	4.67 days

Emerging trends and future directions

The field of robotic-assisted technologies has revolutionized the way certain tasks are performed. One of the most significant trends in robotic-assisted technologies is the seamless integration of artificial intelligence. The integration of AI algorithms enhances the capabilities of robots by enabling them to learn, adapt, and make decisions in real time. This integration allows robots to perform complex tasks with greater efficiency and accuracy. Machine learning algorithms also contribute to improving human-robot interaction, making robots more intuitive and responsive to user needs. However, there is currently no proof that AI can recognize the critical tasks of robotic-assisted surgeries that determine patient outcomes. So, there is a need for studies on large data sets and external authentication of the use of AI algorithms in robotic-assisted surgeries [65]. 

An increase in the amount of autonomy in robotic surgery has the possibility to standardize the surgical outcomes, which are independent of training, experience, and the day-to-day performance changes of the surgeon. The results of a survival study also showed that the developed robotic system can match the performance of an expert surgeon [66]. Robotic-assisted surgeries have not yet been explored in emergency settings, though some of the early experience has been conveyed in the literature [67]. 

Swarm robotics involves the coordination of multiple robots working collaboratively to accomplish tasks. Inspired by the collective behavior of social insects, swarm robotics is gaining traction as a solution for tasks that require scalability, redundancy, and adaptability [68]. With the increasing prevalence of robotics in various industries, the development of ethical guidelines and regulatory frameworks is crucial. As robots become more autonomous and involved in decision-making processes, addressing ethical concerns surrounding their use becomes paramount [69].

The two ongoing areas of research include microrobotics and telesurgery. Samples of investigative micro-robotics are portable capsule endoscopes that deal with various diagnostic tasks, surgical applications, and targeted drug delivery. Mirco-robots are a millimeter in size, with porcine models, and are directed with extracorporeal magnets to apply a particular functional nitinol clip and to stop chronic bleeding during a biopsy [70]. Research is ongoing about the four classes explicit to micro-robotics: miniature functionality, contained propulsion, consistent visualization, and accurate telemanipulation [71].

Further research is also being performed to address the cost, accessibility, safety to the patient, the potential role of fluorescence, and the creation of 3D tracking. The substantial advancement can create another major standard shift in minimally invasive surgery. The new 5G network installed by telecom companies worldwide offers the potential for rapid communication and telesurgery to improve patient's access to quality care and reduce healthcare costs. Telesurgery has been performed in Spain, Italy, Germany, and China with promising results. Studies have also determined that a < 400 ms lag time of imperceptible to the surgeon, and this can be attained by the use of 5G networks [72,73]. Remote nephrotomy was also performed with a median distance of 187km between the patient and surgeon without any conversion and complication. Over time, the feasibility and extent of telesurgery will continue to be tested [70].

Safety and training methods

Despite the increasing interest in the utilization of robotic technology in surgery, there has been a significant increase in the adverse effects related to this approach. The scientific community is engrossed in the need for novel and effective training programs that are capable of preparing adequate robotic surgeons. There are guidelines for open surgery and laparoscopic surgery training, but no guidelines are presented for training in robotic surgeries. Hence, standard, valid, effective, and structured curricula were necessary to have credited and licensed robotic surgeons. This can reduce the exposure of patients to the potential risks [74].

Robotic surgical approaches require a higher level of experience to ensure patient safety. This means that for some surgical procedures, the learning process should be longer than originally expected. As robotic tools progress, a surgeon has to be focused on the machinery type as well as on novel surgical techniques. From a clinical point of view, surgical training programs need to have innovative robotics-assisted surgeries [75]. For this purpose, various validated and standardized training programs have been developed. The European Association of Urology Section (ERUS) has developed the first authenticated and structured curriculum that focuses on robotic-assisted radical prostatectomy in urology [76]. The World Health Organization created the Patient Safety in Robotic Surgery project (SAFROS). The motive of this project is to analyze the safety of robotic surgery, establish safety procedures, and formalize safety requirements and verification protocols [77].

Robotic training curriculums assess the surgical trainee's basic robotic surgical skills or specific protocols using tools including robotic objective structured assessment, technical skills, and global skills assessment [78]. The training curriculum for robotic-assisted surgeries must include theoretical training such as case observation and e-learning, preclinical simulation-based training such as dry lab, wet lab, and virtual reality simulation, clinical modular training, and the final evaluation process. Existing validated curricula, such as the Fundamental Skills of Robotic Surgery, Proficiency-Based Robotic Curriculum, and the Basic Skills Training Curriculum of the Society of European Robotic Gynecological Curriculum, provide valuable frameworks. Despite the availability of various training methods, many validated curricula lack provisions for a monitored modular clinical training phase following the wet and dry lab or virtual reality simulation stages [74]. 

## Conclusions

In summary, the robotic revolution in surgery has ushered in a new era of precision, efficiency, and improved patient outcomes across diverse medical specialties. The advancements in robotic technology have transcended traditional surgical boundaries, enabling minimally invasive procedures with enhanced dexterity and visualization. From general surgery to urology, gynecology, and beyond, robots have become indispensable tools for surgeons, offering unparalleled capabilities in navigating complex anatomical structures. The evolution of robotic-assisted surgery has not only reduced patients' postoperative pain and recovery times but has also expanded the scope of what is surgically achievable. As we look to the future, ongoing research and development promise even greater innovations, potentially paving the way for autonomous surgical systems and further integration of artificial intelligence. Despite challenges, the robotic revolution stands as a testament to the relentless pursuit of excellence in medicine, holding the potential to redefine the landscape of surgical practice in the years to come. The integration of robotic-assisted surgery has highlighted the need for the development of specialized training programs. Surgeons require new skills, such as hand-eye coordination and three-dimensional visualization and interpretation, to operate robotic platforms effectively. Training programs and courses play a crucial role in providing surgeons with the necessary knowledge and skills through lectures, simulation-based training, and hands-on experience. Ongoing education is essential to ensure surgeons stay updated with advancements in the field and optimize patient care. These training programs are vital in equipping surgeons with the expertise needed to navigate the complexities of robotic-assisted surgery.
